# Concurrent Choledochocele and Wirsungocele Presenting With Recurrent Acute Pancreatitis in a 21-Year-Old Male

**DOI:** 10.7759/cureus.113090

**Published:** 2026-07-21

**Authors:** Kenza El Amrani, Fatima Belabbes, Nada Faquir, Hanane Delsa, Imane Ben El Barhdadi

**Affiliations:** 1 Gastroenterology and Hepatology, Mohammed VI University of Sciences and Health, Casablanca, MAR; 2 Gastroenterology and Hepatology, Cheikh Khalifa International University Hospital, Casablanca, MAR

**Keywords:** choledochocele (todani type iii), endoscopic ultrasonography (eus), pancreaticobiliary maljunction, recurrent acute pancreatitis, wirsungocele

## Abstract

Choledochal cysts, including choledochocele (Todani type III), and wirsungocele-cystic dilatation of the terminal main pancreatic duct are uncommon; their coexistence is exceptional and may predispose to pancreatitis. We report the case of a 21-year-old man with recurrent acute pancreatitis who presented with epigastric pain radiating to the back and vomiting. Serum lipase was 611 U/L (~13× the upper limit of normal), with mild cholestatic enzyme elevation. Ultrasound showed acalculous cholecystitis and a dilated common bile duct. A CT scan demonstrated grade C pancreatitis, and magnetic resonance cholangiopancreatography (MRCP) found a biliary tract dilatation with a saccular ectasia at the biliopancreatic junction. Endoscopic ultrasonography (EUS) identified a 3-mm intra-ampullary cyst of the main pancreatic duct (wirsungocele) and a choledochocele. During laparoscopic cholecystectomy, intraoperative endoscopic retrograde cholangiopancreatography (ERCP) with infundibulotomy and biliary sphincterotomy exposed both orifices; balloon sweeping retrieved debris. No stent was placed. The postoperative course was uneventful, symptoms resolved, and liver enzymes normalized at one-month follow-up. This case highlights that dual duct anomalies should be considered in young patients with recurrent "idiopathic" pancreatitis. EUS and ERCP can outperform MRCP for defining small intra-ampullary cysts and enable targeted endoscopic therapy. Given the malignancy risk associated with choledochal cysts, ongoing surveillance is advised.

## Introduction

The common bile duct and main pancreatic duct normally join within the duodenal wall, forming a short common channel that opens at the major papilla under the control of the sphincter of Oddi. A choledochal cyst is a congenital dilatation of the biliary tree. The existing classification system, as outlined by Todani et al. in 1977 [1], categorizes choledochal cysts into five types.

Wirsung cyst or wirsungocele, as designated by Baron et al., is a dilatation of the terminal portion of the main pancreatic duct [2]. These rare conditions are often asymptomatic and discovered fortuitously on imaging [3].

When symptomatic, the symptoms may vary according to age: biliary or pancreatic symptoms and abdominal pain are more commonly observed in adults, while children are more likely to present with jaundice and the presence of an abdominal mass [4].

The coexistence of choledochal and Wirsung cysts in a single patient is exceedingly rare; only a few cases have been reported in the literature. Both anomalies can share a common pathophysiological pathway: an abnormally long common channel and sphincter of Oddi dysfunction allow pancreatobiliary reflux, which triggers pancreatitis. This mechanism also underlies pancreaticobiliary maljunction (PBM).

We report the case of a 21-year-old patient with recurrent acute pancreatitis, in whom both choledochal and Wirsung cysts were diagnosed concurrently.

## Case presentation

A 21-year-old male with a history of acute pancreatitis four months earlier was admitted to the emergency department for acute abdominal pain, which started two days before. The pain was localized in the epigastric region with radiation to the back and was accompanied by vomiting. No jaundice or other signs of cholestasis were reported.

Upon physical examination, no abnormalities were detected. However, laboratory tests revealed significantly elevated lipase levels (13 times the normal range), and liver enzymes were found to be twice the normal levels (Table 1).

**Table 1:** Admission laboratory results. AST: Aspartate aminotranferase; ALT: Alanine aminotransferase; GGT: Gamma-glutamyl transferase; ALP: Alkaline phosphatase; TB: Total bilirubin

Laboratory work-up	Patient's results	Normal range
Lipase (U/L)	611	13-60
AST (U/L)	126	<40
ALT (U/L)	103	<55
GGT (U/L)	293	<45
ALP (U/L)	113	45-132
TB (mg/dL)	2.68	<1

The abdominal US found an acalculous cholecystitis and dilation of the common bile duct. A CT scan demonstrated grade C pancreatitis, with peripancreatic fat stranding but no fluid collection.

Magnetic resonance cholangiopancreatography (MRCP) revealed cholecystitis, peripancreatic inflammatory changes consistent with acute pancreatitis, and dilation of the intrahepatic biliary tract. The common bile duct measured 8.5 mm, with a pseudo-saccular ectasia at the junction of the main bile duct and pancreatic duct.

**Figure 1:**
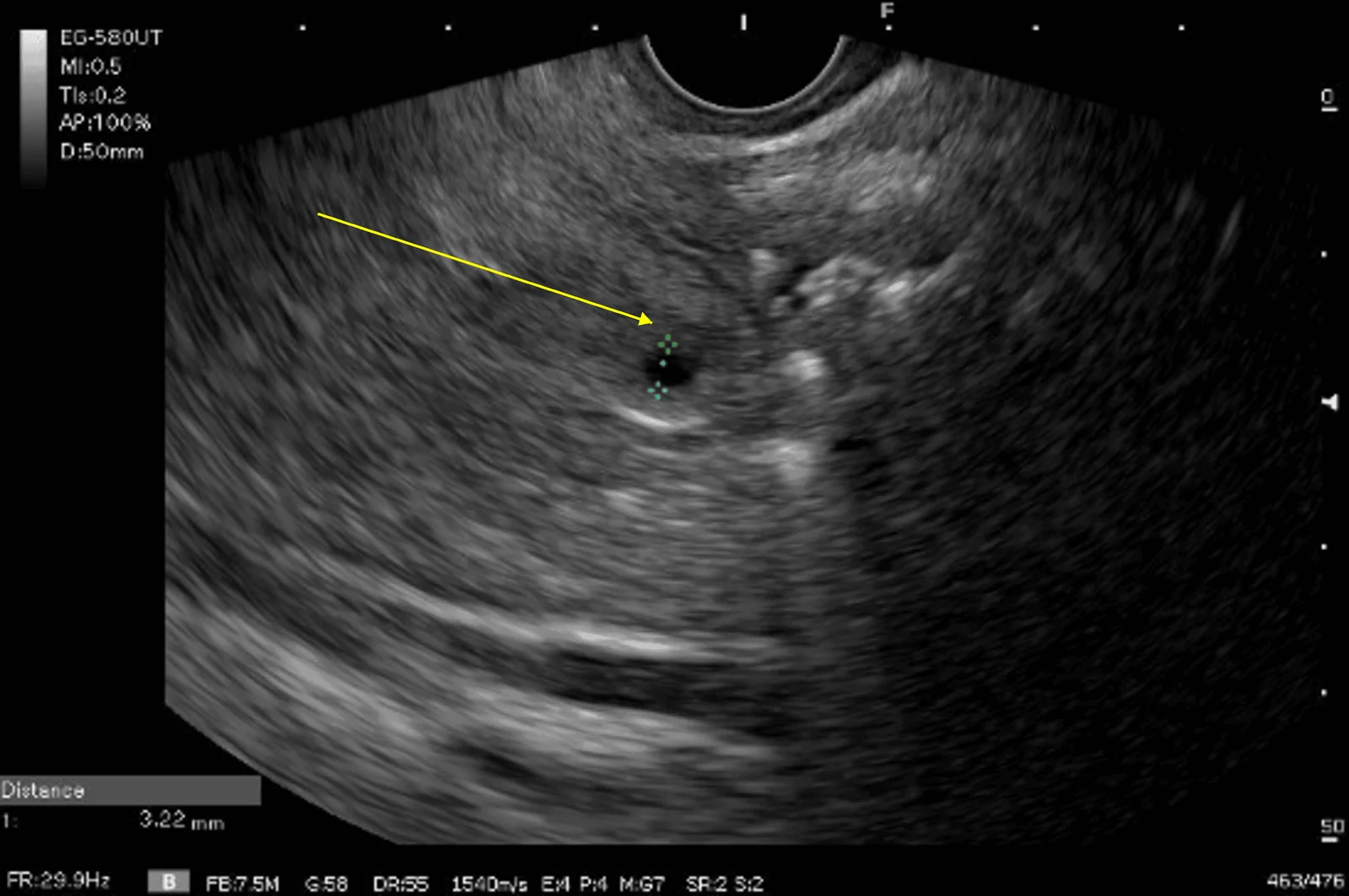
Endoscopic ultrasonography image showing a 3-mm intra-ampullary cystic lesion (arrow), corresponding to the wirsungocele.

Endoscopic ultrasonography (EUS) was performed to determine the cause of the pancreatitis and the nature of the pancreaticobiliary maljunction. It revealed a choledochal cyst and an intra-ampullary Wirsung duct cyst (Figure 1).

Our patient underwent laparoscopic cholecystectomy and intraoperative endoscopic retrograde cholangiopancreatography (ERCP). Cystic duct and standard papillary cannulation failed; a needle-knife fistulotomy was performed at the bulging infundibulum, followed by biliary sphincterotomy and infundibulotomy, exposing both the biliary and pancreatic orifices. Balloon sweeping of the bile duct with an 8-mm balloon retrieved debris without evidence of stones. No stent was placed.

We encountered no complications. The evolution was favorable: the patient did not complain of any pain, vomiting, or digestive hemorrhage, and oral alimentation was tolerated. He was discharged three days after the procedure.

At the one-month follow-up consultation, the patient was asymptomatic, and the laboratory exam showed normal levels of hepatic enzymes.

## Discussion

Santoriniceles, or dilations of the Santorini duct, especially in cases of pancreas divisum, are associated with recurrent acute pancreatitis episodes. Santorinicele affects the accessory pancreatic duct (duct of Santorini), which typically drains through the minor papilla, whereas a wirsungocele involves the main pancreatic duct (duct of Wirsung) at its terminal, intra-ampullary segment. This anatomical distinction explains why a wirsungocele, unlike a santorinicele, directly implicates the major papilla and the biliopancreatic junction, placing it in closer anatomical and pathophysiological proximity to choledochal malformations and PBM.

**Figure 2:**
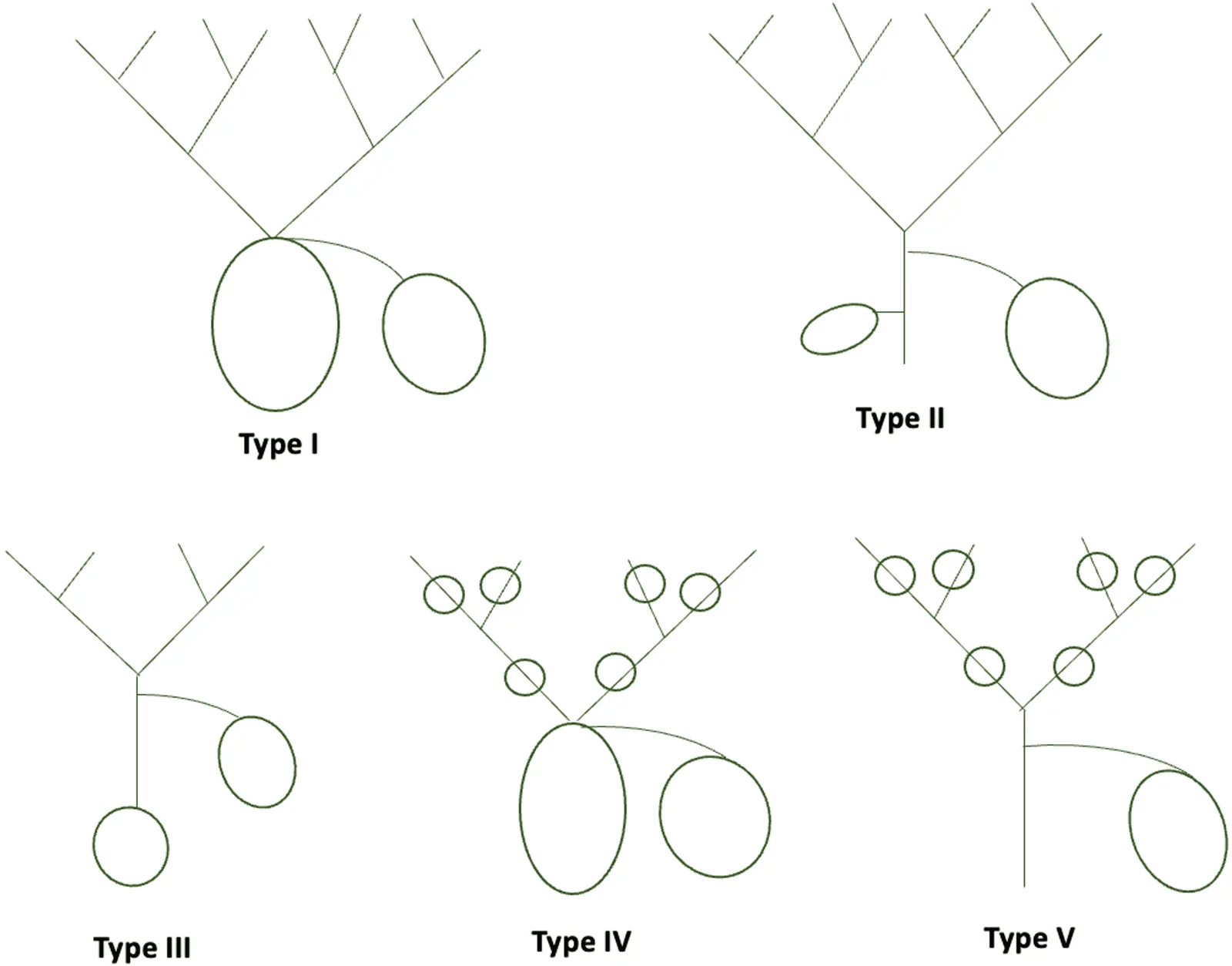
Todani classification. Image credits: K. El Amrani, using Microsoft PowerPoint 2019 (Microsoft® Corp., Redmond, WA), adapted from Todani et al. [5].

All segments of the biliopancreatic tree can be susceptible to cystic malformations. Choledochal malformations are classified by Todani according to localization (Figure 2) [5].

We also observe some instances of recurrent pancreatitis revealing choledochal cysts. On the other hand, symptomatic wirsungoceles are less commonly described in the literature.

Although the exact cause of these cysts remains uncertain, the prevailing theory centers on an abnormal junction between the pancreatic and biliary ducts, referred to as anomalous pancreatico-biliary duct junction (APBDJ) or pancreaticobiliary maljunction (PBM). This condition results in the formation of a lengthy common channel. As a result, the sphincter of Oddi's function is disrupted, leading to the occurrence of pancreaticobiliary reflux, causing cholangitis and dilation of the common bile duct. Additionally, it can affect the pancreatic duct, potentially leading to pancreatitis [6]. Junctional anomalies underlying PBM are further characterized by the Komi classification, which distinguishes three types according to the angle of ductal union: type I (bile duct joining the pancreatic duct at a right angle), type II (pancreatic duct joining the bile duct at an acute angle), and type III (complex junction fitting neither pattern) [7]. The pseudo-saccular ectasia observed at the biliopancreatic junction in our patient, with bidirectional cystic dilatation and free communication between both ducts, corresponds to a complex-type junction rather than a simple right- or acute-angle union. The inflammation can lead to epithelial injury, mucosal dysplasia, and eventually, malignancy.

Because the clinical presentation is often nonspecific, establishing the diagnosis depends primarily on imaging. ERCP is commonly considered the gold standard for diagnosing choledochal cysts and related abnormalities.

Alternatively, MRCP serves as a valuable noninvasive tool with high overall accuracy in detecting and classifying choledochal cysts and may offer comparable diagnostic capabilities to ERCP [8].

A less invasive alternative to ERCP is EUS, which represents a valuable modality for identifying anomalies, as well as complications [9,10].

In our case, EUS and ERCP were significantly more sensitive than MRCP, identifying a 3-mm intra-ampullary cyst that MRCP had failed to characterize as a distinct structure. This illustrates a diagnostic pitfall: MRCP may show only nonspecific junctional dilation, and a normal or borderline MRCP should not exclude a wirsungocele in patients with recurrent pancreatitis.

Surgery is the established treatment for PBM. Prophylactic cholecystectomy is recommended, given the substantial risk of developing gallbladder carcinoma [11]. On the contrary, there is no curative treatment available for the underlying cause of PBM, as pancreaticoduodenectomy is an extremely aggressive and high-risk procedure, making it unsuitable in this context [12].

The management of choledochal cyst varies according to its type, size, and risk of complications. Management of type I, II, and IV-A choledochal cysts typically includes complete extrahepatic bile duct cyst resection extending to the pancreatobiliary junction, cholecystectomy, and restoration of bile flow via a bilioenteric anastomosis.

Considering the low risk of malignant transformation in types II and III, the appropriate management of small choledochoceles consists of endoscopic sphincterotomy [13,14].

Regarding the treatment of the Wirsung cyst, the recommended course of action is endoscopic pancreatic sphincterotomy and placement of a temporary stent that can be removed after four weeks [15,16].

In our case, treatment was cholecystectomy and biliary sphincterotomy. Pancreatic duct stenting was not performed; balloon dilation alone was judged sufficient to relieve the outflow obstruction. Without proper management, these malformations can lead to malignant transformation.

A meta-analysis conducted by Ten Hove et al. found that, out of the 2,904 cases of CC, 11% developed cancer [17].

In patients with choledochal cysts, malignant lesions are most often adenocarcinomas, representing about 73%-84% of cases, followed by anaplastic carcinomas in roughly 10%, undifferentiated carcinomas in 5%-7%, squamous cell carcinomas in about 5%, and other, less common histological types accounting for approximately 1.5% of cases [18].

It is most commonly observed in patients with CCs of type I (accounting for 68% of cases) and type IV (accounting for 21% of cases) [19].

According to published data, CC type IV carries a malignancy rate of 21%, with corresponding rates of 5% for type II, 1.6% for type III, and 6% for type V [20].

As for wirsungoceles, we did not find any reports of malignant transformation. This could be due to the limited number of cases of wirsungoceles described in the literature.

## Conclusions

Coexisting choledochocele (Todani III) and wirsungocele is rare and may present with recurrent acute pancreatitis in young adults. In this patient, EUS and ERCP provided a more detailed assessment of the small intra-ampullary lesions than MRCP, enabling targeted endoscopic therapy with symptom resolution at one month. Management should be individualized according to cyst type and ampullary anatomy. Choledochal cysts warrant ongoing surveillance, given their risk of malignancy, which persists even for Todani type III, despite its comparatively lower rate. In contrast, malignant transformation of a wirsungocele has not been reported to date. Regular follow-up with MRI or EUS is therefore recommended even after symptom resolution.

This case shows that dual pancreatobiliary anomalies should be a key consideration in presumed idiopathic pancreatitis, and a low threshold for utilizing EUS is recommended.
